# On the genetic structure and origin of the little ground squirrel Spermophilus pygmaeus (Pallas, 1778) in the North Caucasus

**DOI:** 10.18699/vjgb-25-87

**Published:** 2025-10

**Authors:** F.A. Tembotova, A.Kh. Amshokova, M.S. Gudova

**Affiliations:** Tembotov Institute of Ecology of Mountain Territories of the Russian Academy of Sciences, Nalchik, Russia; Tembotov Institute of Ecology of Mountain Territories of the Russian Academy of Sciences, Nalchik, Russia; Tembotov Institute of Ecology of Mountain Territories of the Russian Academy of Sciences, Nalchik, Russia

**Keywords:** Spermophilus pygmaeus, cytochrome b (cyt b), genetic diversity, Western Caucasus, Spermophilus pygmaeus, cyt b, генетическое разнообразие, Западный Кавказ

## Abstract

Little ground squirrel Spermophilus pygmaeus (Pallas, 1778) is a polytypic species of significant interest for the study of taxonomic diversity, genetic structure, gene flow and genetic diversity. Despite the long history of study, the taxonomy of representatives of the genus Spermophilus in the North Caucasus remains poorly developed. Among the unresolved issues are the phylogenetic relationships between the “mountain” and “plain” ground squirrels of the North Caucasus. An equally important aspect of the work is the study of the genetic diversity of little ground squirrel, given that the species is considered an integral component of steppe and desert ecosystems, providing their most important biocenotic functions. Based on the analysis of the 840 bp mtDNA cytochrome b gene fragment, new data on the genetic variability of S. pygmaeus from the eastern extremity of the Western Caucasus were obtained. Unlike previous studies that showed the so-called mountain ground squirrel to inhabit the Caucasus Mountains, this work identified two haplogroups of S. pygmaeus in the studied areas at an altitude of 1,400–1,700 m above sea level, one of which is close to the lowland (East Caucasian) and the other to the mountain (Central Caucasian) groups of the little ground squirrel. The genetic distance between the two haplogroups was 1.54 %. The different evolutionary ages of the three identified groups of S. pygmaeus in the North Caucasus (A1, A2, and B) are most likely associated with the multi-stage settlement of the studied area by the little ground squirrel. The results of molecular dating suggest that the western haplogroup penetrated as a continuous strip into the Central, Eastern Caucasus and the eastern extremity of the Western Caucasus through the Stavropol Upland and the Caspian Lowland less than 400 thousand years ago. As a result of the first wave of dispersal of the ground squirrel from the Russian Plain, the species became established in the eastern extremity of the Western Caucasus in the area of the village of Khasaut, and in the Eastern Caucasus – in the north of the Nogai Steppe (Sukhokumsk) and in the southern outskirts of the Caspian Lowland (Kar-Kar 1 Valley). The younger age of haplogroup A2 (less than 300 thousand years), also originating from the Eastern Caucasus (Khumtop, Zelenomorsk, Lvovsky 13, Kar-Kar 2), is most likely due to the re-colonization of the Caspian lowland by the ground squirrel, which was regularly flooded by the Caspian Sea in historic times. The absence of a continuous forest belt in the Central Caucasus, in particular in the Kabardino-Balkarian Republic, allowed S. pygmaeus to penetrate into the mountains later, less than 200 thousand years ago, through three gorges: Cherek, Baksan and Malkinsky. It is more likely that the species penetrated into the subalpics of the Western Caucasus (Khurzuk and Uchkulan) from the Central Caucasus, as evidenced by the same evolutionary age of animals of the Western (Uchkulan, Khurzuk) and Central Caucasus. Regarding the taxonomic status of the Caucasian mountain ground squirrel, we consider it premature to draw any conclusions, since not all areas of the Caucasus were covered by research.

## Abstract (ru)

Малый суслик Spermophilus pygmaeus (Pallas, 1778) – политипический вид, представляющий существенный интерес для изучения таксономического разнообразия, генетической структуры, потока генов и генетического разнообразия. Несмотря на длительную историю изучения, систематика представителей рода Spermophilus на Северном Кавказе остается слабо разработанной. Неразрешенным вопросом остаются и филогенетические отношения между «горными» и «равнинными» сусликами Северного Кавказа. Не менее важным аспектом работы является оценка генетического разнообразия малого суслика, поскольку вид считается неотъемлемым компонентом степных и пустынных экосистем, обеспечивая их важнейшие биоценотические функции. На основании анализа фрагмента гена cyt b мтДНК длиной 840 п. н. получены новые данные по генетической изменчивости Spermophilus рygmaeus восточной оконечности Западного Кавказа. В отличие от ранее проведенных работ, показавших обитание в горах Кавказа так называемого горного суслика, в настоящей работе на исследованных территориях на высоте 1400–1700 м над ур. моря выявлены две гаплогруппы S. pygmaeus, одна из которых близка к равнинным (восточно-кавказским), а вторая – к горным (центрально-кавказским) группировкам малого суслика. Генетическая дистанция между двумя гаплогруппами составила 1.54 %. Разный эволюционный возраст трех выявленных группировок S. pygmaeus на Северном Кавказе (A1, A2 и B), скорее всего, связан с многоэтапным заселением исследуемой территории малым сусликом. Данные молекулярного датирования позволяют предположить, что западная гаплогруппа проникла сплошной полосой на Центральный, Восточный Кавказ и восточную оконечность Западного Кавказа через Ставропольскую возвышенность и Прикаспийскую низменность менее 400 тыс. лет назад. В процессе первой волны расселения суслика с Русской равнины вид закрепился в восточной оконечности Западного Кавказа в районе с. Хасаут, а на Восточном Кавказе – на севере Ногайской степи (Сухокумск) и в южных окраинах Прикаспийской низменности (долина Кар-Кар 1). Более молодой возраст гаплогруппы A2 (менее 300 тыс. лет), также происходящей с Восточного Кавказа (Хумтоп, Зеленоморск, Львовский 13, Кар-Кар 2), вероятнее всего, обусловлен повторным заселением сусликом Прикаспийской низменности, регулярно затапливаемой водами Каспия в историческое время. Отсутствие сплошного пояса лесов на Центральном Кавказе, в частности в Кабардино-Балкарии, позволило позже, менее 200 тыс. лет назад, проникнуть S. pygmaeus в горы по трем ущельям: Черекскому, Баксанскому и Малкинскому. Более вероятно, что в субальпику Западного Кавказа (Хурзука и Учкулана) вид проник уже с Центрального Кавказа, о чем свидетельствует один и тот же эволюционный возраст животных Западного (Учкулан, Хурзук) и Центрального Кавказа. Касательно таксономического статуса кавказского горного суслика считаем преждевременным делать какие-либо выводы, так как не все территории Кавказа были охвачены исследованиями.

## Introduction

The little ground squirrel Spermophilus pygmaeus (Pallas, 1778) is a polytypic species of considerable interest for the study of taxonomic diversity, genetic structure, gene flow, and genetic diversity. Despite the long history of study, the taxonomy of representatives of the genus Spermophilus in the North Caucasus remains poorly developed. The mountain ground squirrel was first collected on the northern slope of Elbrus in the subalpine meadow belt and was described by E. Menetries as an independent species (Menetries, 1832). Many researchers distinguished the mountain ground squirrel as an independent species (Brandt, 1843; Sviridenko, 1937; Vinogradov, Argiropulo, 1941; Mammalian Fauna…, 1963; Fauna of the USSR, 1965; Vorontsov, Lyapunova, 1969; Gromov, Baranova, 1981; Korablev, 1983; Harrison et al., 1993; Hoffmann, 1993; Gromov, Erbaeva, 1995; Tsvirka et al., 2003; Tsvirka, Korablev, 2014), while other authors believe that the Caucasian mountain ground squirrel is a subspecies of the little one (Satunin, 1907; Obolenskii, 1927; Ognev, 1947; Vereshchagin, 1959; Orlov et al., 1969; Ivanov, 1976; Ermakov et al., 2006; Nikol’skii et al., 2007). I.Ya. Pavlinov and A.A. Lisovsky (2012) distinguish Spermophilus (pygmaeus) pygmaeus (left-bank little ground squirrel relative to the Volga River), Spermophilus (pygmaeus) planicola (right-bank little ground squirrel) and Spermophilus (pygmaeus) musicus (Caucasian (mountain) little ground squirrel) as subspecies in the superspecies “pygmaeus”. This species has attracted the attention of researchers for a long time, but there are very few works based on the analysis of the cyt b gene region of the little ground squirrel in the designated area (Harrison et al., 1993; Ermakov et al., 2023; Tembotova et al., 2024). Among the unresolved issues, as rightly noted by O.A. Ermakov and co-authors, are the phylogenetic relationships between the “mountain” and “plain” ground squirrels of the North Caucasus (Ermakov et al., 2018). Also, questions concerning the evolutionary history, the patterns of distribution of the plain and mountain forms of the little ground squirrel are still not fully clarified. The solution of the above-mentioned issues was hampered by the extremely uneven study of the territory with the involvement of insignificant samples in the analysis

All populations of the little ground squirrel living west of the Volga to the lower Dnieper, as well as in Crimea and the Ciscaucasia, were assigned to the sister species S. musicus Ménétries, 1832, since it is the senior synonym applicable to the western lineage of S. pygmaeus sensu lato (Simonov et al., 2024). Based on the noted work, S. musicus is not only the mountain ground squirrel, but also all little ground squirrels of the right bank of the Volga. This raises the question of whether all right-bank little ground squirrels are genetically homogeneous. The emergence of this question is associated with the results we obtained earlier based on the analysis of the mtDNA cytochrome b gene fragment of ground squirrels of the Eastern and Central Caucasus (Tembotova et al., 2024).

The study revealed that two genetically distinct groups of little ground squirrels inhabit the territory of the Eastern Caucasus. In addition, a comparison of the Central Caucasian (mountain) and East Caucasian (plain) groups of little ground squirrels revealed a genetic distance of 1.34 % and an absence of identical haplotypes in the compared groups, which generally indicates the genetic heterogeneity of S. pygmaeus in the studied areas. Distances of the same order (1.29–1.72 %) were obtained between mountain little ground squirrels and ground squirrel populations from the right bank of the Volga River. At first glance, the obtained distances may seem insignificant, but they reach the lower limits of interspecific differences when compared with the distances obtained for representatives of the genus Spermophilus: a minimum of 1.4 % (between the species S. major and S. selevini (=S. brevicauda)) and a maximum of 10.7 % (between S. dauricus and S. xanthoprymnus (Simonov et al., 2024)). It is also not entirely clear whether all populations of the so-called Caucasian mountain ground squirrel inhabiting the mountainous territories of the Caucasus are genetically close to each other and differ equally from the lowland ones.

It should be noted that in most studies attempting to clarify the taxonomic status of the Caucasian mountain ground squirrel, the material was studied mainly only from the Central Caucasus, in particular from the vicinity of Elbrus (Harrison et al., 1993; Ermakov et al., 2006; Nikol’skii et al., 2007; Frisman et al., 2014; Tsvirka, Korablev, 2014), Baksan, Dzhily-Su, Shadzhatmaz gorges (Ermakov et al., 2023). The absence of both literary data obtained on the basis of analysis of the cytochrome b gene region of the little ground squirrel of the Western Caucasus and sequences deposited in the GenBank database determines the need to study samples of the little ground squirrel from this territory to determine the status of S. pygmaeus inhabiting mountainous areas.

Mitochondrial DNA is one of the most frequently used genetic markers in phylogeographic studies of vertebrates (Avise, 2000; Kholodova, 2009; Lukashov, 2009), which is due to such properties as maternal inheritance, an absence of the recombination process, a high rate of evolution compared to nuclear genes, a large number of copies, etc. Cytochrome b has proven itself to be informative and is successfully used in theriological studies at levels from generic to intraspecific (Bannikova, 2004; Abramson, 2007; Kholodova, 2009). In addition, this is the gene for which the most information is available in genetic databases.

The second, no less important, aspect of the work is the study of the genetic diversity of geographic samples of the little ground squirrel. It is known that ground squirrels are an integral component of steppe and desert ecosystems, providing their most important biocenotic functions, but since ancient times they have attracted attention mainly as agricultural pests and carriers of various diseases. Since the 1920s, the fight against ground squirrels as agricultural pests has been going at the state level. Grandiose extermination work against little ground squirrels was carried out in the arid landscapes of the former USSR, natural foci of plague. In the first half of the 20th century, the concept of complete elimination of foci of this infection by extermination of rodents – the carriers of the pathogen – was developed. In the North Caucasus and the North-West Caspian region, almost complete destruction of little ground squirrels was recommended (Kalabukhov, 1933; Pastukhov, 1959). The “recovery” of the natural focus began in accordance with a special program, which reached an unprecedented scale (cited by Shilova, 2011). As a result, the continuous clearing of the territory from the ground squirrel, plowing of the steppes, changes in the intensity of livestock grazing, the development of forest shelterbelts and artificial irrigation led to the destruction of gophers not only in the Caucasus, but also in many regions of Russia. According to S.A. Shilova (2011), a deep depression in the population of the right-bank little ground squirrel began in the south of Russia at the end of the last century and continues to this day. In Kabardino-Balkaria, agricultural development of plain and foothill territories led to an almost complete disappearance of little ground squirrel populations in these territories. In particular, foothill and plain populations of S. pygmaeus are listed in the Red Book of the Kabardino-Balkarian Republic (2018) with the status of “on the verge of extinction”.

It should be noted that habitat transformation and fragmentation due to human activities resulted in a reduction in the areas suitable for little ground squirrel habitation, which led to an extremely high fragmentation of the species populations. At the same time, it is known that fragmentation and reduction of ranges often affect the genetic structure of wild animal populations, complicating the exchange of genes between different parts of the range, reducing the effective population size and leading to an increase in the level of inbreeding.

In connection with the above, the aim of the work was to study the genetic structure and genetic diversity of S. pygmaeus from the eastern end of the Western Caucasus based on the analysis of the mtDNA cytochrome b gene fragment. The results of this study were compared with the ones previously obtained (Ermakov et al., 2023; Tembotova et al., 2024) in order to assess the taxonomic diversity of S. pygmaeus in the North Caucasus.

## Materials and methods

**Fig. 1.**
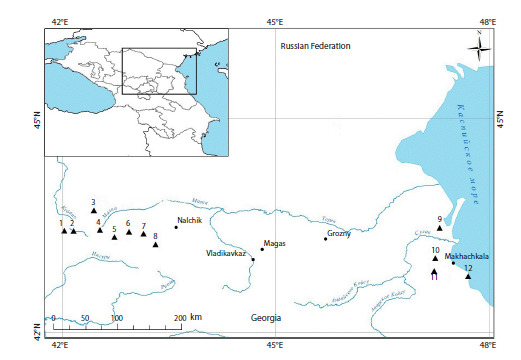
Material origin card of S. pygmaeus in the North Caucasus. Western Caucasus (KCR): 1 – Uchkulan, 2 – Khurzuk, 3 – Khasaut (new data); Central Caucasus (Kabardino-Balkarian Republic): 4 – Dzhily- Su, 5 – Elbrus, 6 – Tyrnyauz, 7 – Aktoprak, 8 – Bezengi; Eastern Caucasus (Republic of Dagestan): 9 – Lvovsky 13, 10 – Khumtop, 11 – Kar-Kar, 12 – Zelenomorsk (Tembotova et al., 2024).

In this work, we used muscle tissue samples of S. pygmaeus from different geographical locations of the eastern end of the Western Caucasus: Karachay-Cherkess Republic (KCR): upper reaches of the Kuban River, in the vicinity of the villages of Khurzuk and Uchkulan; the Khasaut River tract, a tributary of the Malka River, in the vicinity of the village of Khasaut (Fig. 1). The animals were caught using No. 0 arc traps. The traps were set around the gophers’ residential burrows (Karaseva, Telitsina, 1996).

The analyzed sample included 32 sequences of the mitochondrial cytochrome b gene (cyt b) of S. pygmaeus from the KCR, collected in the vicinity of the village of Khasaut and the villages of Khurzuk and Uchkulan (Table 1). In addition, haplotypes of the North Caucasus little ground squirrel from our previously published work (Tembotova et al., 2024) were also used to conduct a comparative analysis.

**Table 1.**
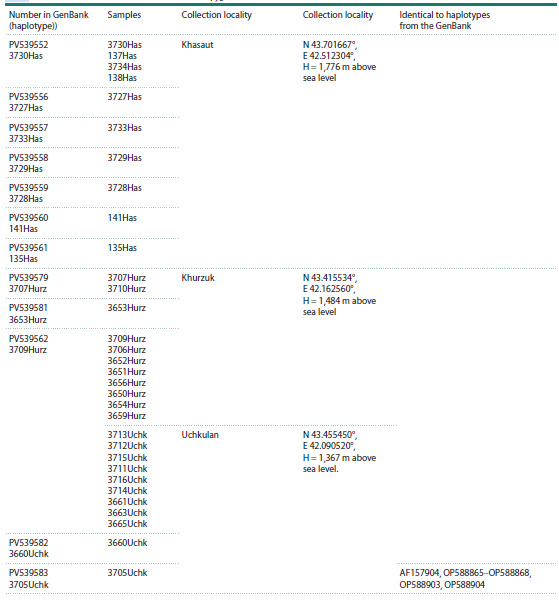
Characteristics of the studied material on S. pygmaeus from the eastern end of the Western Caucasus (KCR)

The remaining sequences of representatives of the genus Spermophilus, including the outgroup, were taken from the GenBank database (ncbi.nlm.nih.gov): S. pygmaeus – OP588846–OP588904 (Ermakov et al., 2023), AF157907, AF157910 (Harrison et al., 1993); S. musicus – AF157900, AF157904 (Harrison et al., 1993), Spermophilus taurensis Gündüz et al., 2007 – KY938064, KY938069, KY938073 (Gür, 2017); Spermophilus сitellus Linnaeus, 1766 – AM691632– AM691640; Spermophilus xanthoprymnus Bennett, 1835 – AM691658–AM691663 (Gündüz et al., 2007) and AF157902, AF157909 (Orlov et al., 1969); Marmota monax Linnaeus, 1758 – AF157953 (Harrison et al., 1993).

DNA extraction was performed using the Diatom™ DNA Prep100 kit (Isogene Laboratory, Moscow) according to the manufacturer’s protocol. DNA fragments were amplified using the MasterMix X5 kit (Dialat, Moscow). The following primers were used for the polymerase chain reaction: L14725 TGAAAAAYCATCGTTGT (Steppan et al., 1999) H15915 TCTTCATTTYWGGTTTACAAGAC (Harrison et al., 1993), with the PCR cycle parameters recommended in the first work. The resulting PCR products were purified by reprecipitation in 0.15 M sodium acetate solution, in 90 % ethanol, followed by washing with 70 % ethanol. The quality of the obtained PCR products was assessed by electrophoresis in 1.5 % agarose gel in the presence of ethidium bromide. Sequencing of nucleotide sequences was performed in both directions at Syntol (Moscow). Editing and alignment of the obtained sequences were performed using the BioEdit 7.0.9.0 program (Hall, 1999) using the Clustal W algorithm and edited manually.

Statistical data processing, including calculation of the number of polymorphic sites, the number of haplotypes, nucleotide and haplotype diversity, as well as the neutrality tests of Tajima (Tajima, 1989) and Fu (Fu, 1996), was performed in the Arlequin v.3.5 program (Excoffier, Lischer, 2010). In the same program, an analysis of the distribution of observed and expected values of pairwise nucleotide differences in mtDNA was carried out in accordance with the models of demographic (Rogers, Harpending, 1992) and spatial expansion (Ray et al., 2003). Weighted (net distance) intergroup genetic distances using the Kimura two-parameter model (K2P) (Kimura, 1980) were calculated in the Mega 6 program.

Median haplotype networks were constructed in the Network 4.6.1 program using the Median-Joining method (Bandelt et al., 1999) and then edited using the standard Paint package.

Phylogenetic analysis of nucleotide sequences using the Bayesian MCMC method was performed in MrBayes v3.2.6. (Ronquist, Huelsenbeck, 2003).

Divergence times were estimated in BEAST 1.10.4 (Suchard et al., 2018) using the following calibrations: 10.9 million years for the root divergence node of Marmota and other Spermophilus species (Yin et al., 2014), 5 million years for the divergence time between S. xanthoprymnus and S. сitellus + S. taurensis, and 2.5 million years between S. сitellus and S. taurensis (Gündüz et al., 2007). Data were analyzed using an uncorrelated lognormal relaxed molecular clock model. The most optimal model of nucleotide substitutions (HKY + I) was selected using the MEGA 6 software package. The length of Markov chains (MCMC, Markov Chain Monte Carlo) was set equal to 100 million generations with the selection of every thousandth state and a burn-in value of 10 %. The convergence of the parameters was assessed based on achieving ESS (effective sample size) values >200 using Tracer 1.7 software (Rambaut et al., 2018). The divergence time of the Spermophilus dendrogram nodes was calculated for six variants of nucleotide substitutions per million years: 0.5, 0.9, 1.2, 2.4, 3.2 and 6.7 %.

## Results

Based on the analysis of tissue samples of S. pygmaeus from the eastern end of the Western Caucasus, 32 nucleotide sequences of the cytochrome b gene fragment with a length of 840 bp were obtained. All of them were uploaded to the GenBank database under the numbers PV539552–PV539583. The analyzed sequences contained 24 variable sites, of which 14 were parsimony-informative.

**Fig. 2.**
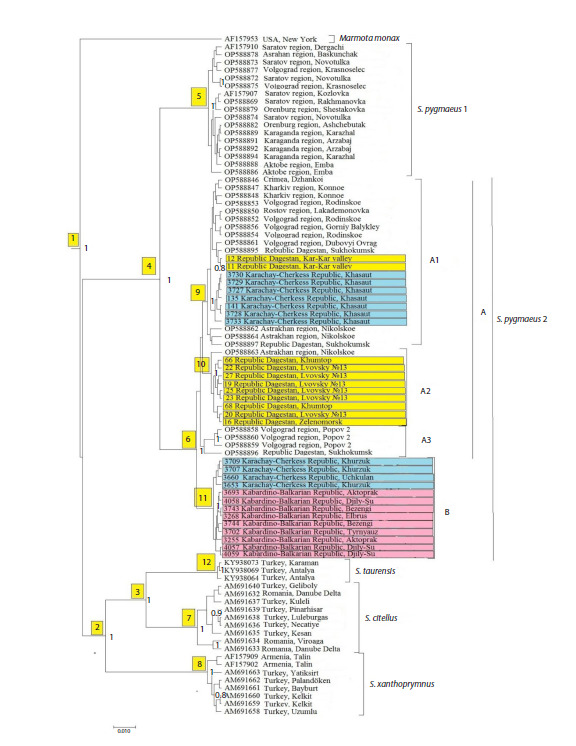
Bayesian phylogenetic tree of representatives of the genus Spermophilus based on the analysis of the mtDNA cyt b gene (840 bp) (after (Tembotova et al., 2024) with additions). The numbers in the branching nodes are the values of posterior probabilities (greater than 0.70), the numbers in the squares are the node numbers. The haplotypes of the little ground squirrel of the Republic of Dagestan are highlighted in yellow, those of the Karachay-Cherkess Republic are highlighted in blue, and those of the Kabardino-Balkarian Republic are highlighted in pink.

The results of the phylogenetic analysis showed the same tree topology (Fig. 2) as in previous studies performed on this group and revealing the division of S. pygmaeus into western and eastern groups (Ermakov et al., 2023).

Previously, we demonstrated the isolated position of the Central Caucasian (mountain) and East Caucasian (plain) samples on the phylogenetic tree (Tembotova et al., 2024). The new haplotypes from the Karachay-Cherkess Republic analyzed in this work were split into two haplogroups. Thus, out of the three analyzed samples, the mitotypes of two samples (Uchkulan and Khurzuk) fell into haplogroup (B), formed by the haplotypes of the Central Caucasian animals. Unlike other mountain samples, little ground squirrels from the vicinity of the village of Khasaut are closer to the plain haplotypes and form one haplogroup A1 with them.

**Fig. 3.**
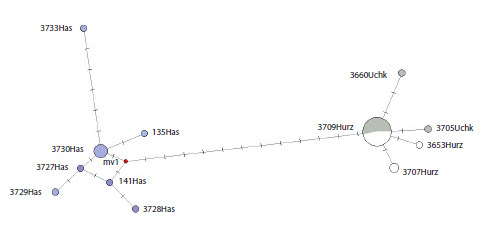
Median network of mtDNA haplotypes of S. pygmaeus, constructed based on the analysis of the cyt b region (algorithm – median-joining, Network 4.6.1). The size of the circle is proportional to the number of identical haplotypes. Haplotypes identified in the territory of the Karachay- Cherkess Republic: Hurz (vicinity of the village Khurzuk) – in white, Uchk (vicinity of the village Uchkulan) – in gray, Has (vicinity of the village Khasaut) – in lilac. The number of cross bars on the branches indicates the number of nucleotide substitutions; the “mv” mark denotes hypothetical haplotypes.

The median network of haplotypes also demonstrates the existing distribution of the haplotypes found in the territory of the Karachay-Cherkess Republic into two main haplogroups (Fig. 3). It is noteworthy that almost all mitotypes from the vicinity of the village of Khasaut form a separate compact haplogroup. The second haplogroup included mitotypes of the little ground squirrel from Uchkulan and Khurzuk. The genetic distance between the two haplogroups was 1.54 %.

The obtained 32 sequences from the KCR formed 12 haplotypes, of which nine were unique, and three were described in 2–17 individuals. As can be seen from Table 1 and Figure 3, the maximum number of unique haplotypes (60 %) is noted in the sample from the vicinity of the village of Khasaut. The most frequently occurring mitotype 3709Нurz was noted in eight individuals from the vicinity of the village of Khurzuk and nine individuals from the vicinity of the village of Uchkulan. For the 22 studied individuals from Uchkulan and Khurzuk, only five haplotypes were described, of which three are unique. It is possible that the loss of haplotypes is associated with a general decrease in numbers

**Fig. 4.**
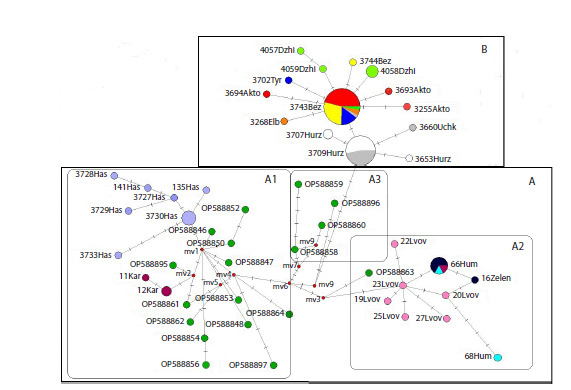
Median network of mtDNA haplotypes of S. pygmaeus, constructed on the basis of the analysis of the cyt b region (algorithm – median-joining, Network 4.6.1). The size of the circle is proportional to the number of identical haplotypes. Haplotypes identified in the Republic of Dagestan: Kar (Kar-Kar Valley) – are shown in brown, Zelen (vicinity of the village Zelenomorsk ) – in black, Hum (vicinity of the village Khumtop) – in blue, Lvov (vicinity of the village Lvovsky 13) – in pink; in Kabardino-Balkarian Republic: Tyr (vicinity of the town Tyrnyauz) – in blue, Bez (vicinity of the village Bezengi) – in yellow, Akto (Aktopraksky Pass) – in red, Elb (vicinity of the village Elbrus) – in orange, Dzhi (Dzhily-Su tract) – in light green; in Karachay-Cherkess Republic: Hurz (vicinity of the village Khurzuk) – in white, Uchk (vicinity of the village Uchkulan) – in gray, Has (vicinity of the village Khasaut) – in lilac. The number of crossbars on the branches indicates the number of nucleotide substitutions; the “mv” mark indicates hypothetical haplotypes.

To clarify the clustering of the analyzed haplotypes, an additional median network was constructed (Fig. 4) with the inclusion in the analysis of sequences of the little ground squirrel previously obtained by us (Tembotova et al., 2024) and O.A. Ermakov et al. (2023).

Analysis of the median network demonstrates the division of the little ground squirrel haplotypes into two groups: the plain (A), which in turn is subdivided into three haplogroups (A1, A2, A3) and the mountain (B). As shown earlier, the sample from the vicinity of the village of Khasaut clusters together with mitotypes from haplogroup A1. Two haplotypes, 11Kar and 12Kar, from the Eastern Caucasus (Republic of Dagestan, Kar-Kar 1 Valley) also fell here. The genetic distance between the animals from the Kar-Kar 1 Valley and those from the vicinity of the village of Khasaut was only 0.36 %. The haplotypes of the animals from Uchkulan and Khurzuk, together with the Central Caucasian samples (Bezengi village, Aktopraksky pass, Tyrnyauz area, Irikchat gorge, Dzhily-Su tract), formed a separate haplogroup B. The most common haplotype 3709Uch from the Karachay-Cherkessia Republic differs from the haplotype 3743Bez, described in 39 individuals from different geographical points of the Central and Western Caucasus, by a single substitution. In addition, it is important to note that the haplotype 3705Uch turned out to be identical to the Central Caucasian haplotype 3743Bez. Haplogroup A2 is formed by the haplotypes of only the East Caucasian animals.

**Table 2.**
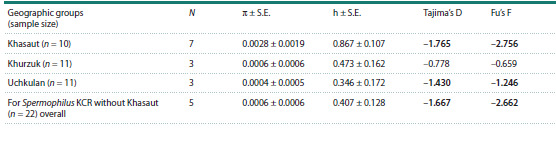
Indicators of haplotype (h) and nucleotide (π) diversity and the values of the Tajima’s and Fu’s tests of S. pygmaeus from Karachay-Cherkess Republic Note. N – number of haplotypes; S.E. – standard error. Statistically significant test values are highlighted in bold.

Analysis of genetic variability showed that little ground squirrel samples from the area near the villages of Uchkulan and Khurzuk are characterized by low values of haplotype (h) and nucleotide (π) diversity (Table 2). In the sample from the vicinity of the village of Khasaut, on the contrary, relatively high values of nucleotide and haplotype diversity are observed. Thus, the nucleotide diversity in this sample was 0.0028, and the haplotype diversity was 0.867. Since the sample from the vicinity of the village of Khasaut is clustered separately from the other samples from the Karachay-Cherkess Republic, the parameters of genetic variability of the populations were calculated only for two combined samples – Uchkulan and Khurzuk. As a result, for the combined sample (n = 22), the haplotype diversity was 0.407 ± 0.128, the nucleotide diversity was 0.0006 ± 0.0006

The values of the Tajima’s D and Fu’s Fs tests in all three samples were negative (Table 2), while the Tajima’s and Fu’s tests were significant for two samples – Uchkulan and Khasaut. In the combined sample (Uchkulan+Khurzuk), the values of the Tajima’s and Fu’s tests were also negative and statistically significant.

Of the three noted little ground squirrel haplogroups (A1, A2 and B), A1 and B are the most genetically distinct, with a genetic distance of 1.46 %. Nearly the same distance (1.41 %) was obtained between haplogroups A2 and B. And finally, the minimum distance was obtained when comparing groups A1 and A2 (0.74 %). Regarding the genetic distances obtained between the three studied samples from the Karachay-Cherkess Republic, it should be noted that when comparing little ground squirrels from Uchkulan and Khurzuk, the obtained distance was zero. And the sample from Khasaut was equally different from the S. pygmaeus groups from Uchkulan and Khurzuk with a distance of 1.53 %

Table 3 shows the genetic distances between the geographic samples of S. pygmaeus from the North Caucasus. As can be seen, the sample from the vicinity of the village of Khasaut differs from all other Central Caucasian samples (Aktoprak, Bezengi, Irikchat, Tyrnyauz, Dzhily-Su) with distances of 1.5–1.7 %, and from the East Caucasian samples, with distances of 0.4–1 %. When comparing two West Caucasian samples (Uchkulan, Khurzuk) with the Central Caucasian samples, the distances were only 0–0.2 %, and when they were similarly compared with the East Caucasian samples, the minimum distance was 1.2 %, and the maximum was 1.7 %.

**Table 3.**
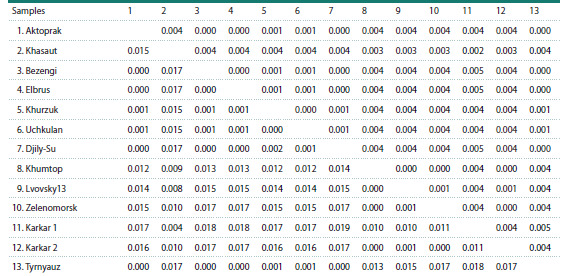
Genetic distances between geographical samples of S. pygmaeus from the North Caucasus (mtDNA cytochrome b gene region) Note. Below the diagonal are the values of intergroup distances, above the diagonal are the corresponding values of the standard error.

**Fig. 5.**
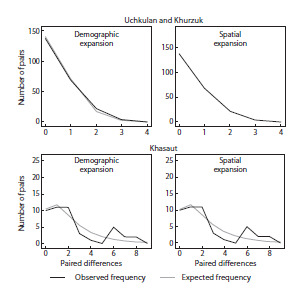
Histograms of the distribution of paired differences (mismatch distribution) of S. pygmaeus: vicinity of the villages of Uchkulan+Khurzuk and vicinity of the village of Khasaut (demographic expansion and spatial expansion).

The analysis of the frequency distribution of paired nucleotide differences between haplotypes (Fig. 5) was also carried out for two samples: from the vicinity of the village of Khasaut and the combined Uchkulan+Khurzuk sample. The combined sample shows a unimodal distribution pattern, close to expectations for a growing population, which may probably indicate a recent demographic expansion or spatial expansion (less than 200 thousand years ago) after a decline in population. The analysis of the distribution of the number of nucleotide substitutions in the sample from the vicinity of the village of Khasaut revealed multimodality, which probably indicates the presence of two or more subpopulations.

As noted earlier (Tembotova et al., 2024), the results of molecular dating were obtained based on three calibration points: 10.9 million years for the root node of divergence of Marmota and other Spermophilus species (Yin et al., 2014), 5 million years for the divergence time between S. xanthoprymnus and S. сitellus + S. taurensis, and 2.5 million years between S. сitellus and S. taurensis (Gündüz et al., 2007). The inclusion of additional samples from the Karachay-Cherkess Republic in the analysis did not significantly affect the previously obtained results (Tembotova et al., 2024), and the age of many nodes remained almost the same (Table 4).

**Table 4.**
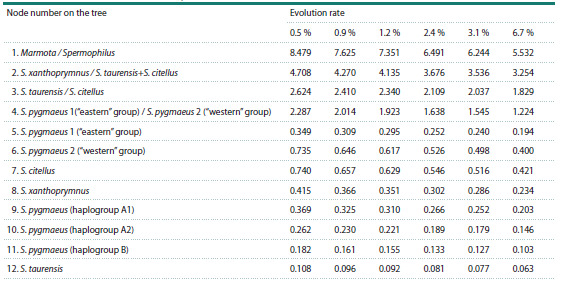
Divergence times (in million years) of Spermophilus taxa and individual clusters with six evolutionary rate variants

As for the samples from the Karachay-Cherkess Republic, the evolutionary age of the little ground squirrel group from Khasaut together with some haplotypes from Dagestan (11, 12, Kar-Kar valley, OP588895, OP588897, Sukhokumsk), as well as Crimea, Kharkov, Volgograd, Rostov, Astrakhan regions was 369 thousand years (95 % HPD: 0.217–0.538 million years) (node 9) for the model calculated for the mutation rate of 0.5 % per million years. The remaining haplotypes of animals from the Karachay-Cherkess Republic (Uchkulan, Khurzuk) fell into the cluster (B) formed by mitotypes of the Central Caucasian animals. The age of this cluster was 182 thousand years (95 % HPD: 0.080–0.300 million years) (node 11).

## Discussion

Based on the obtained 32 sequences, 12 haplotypes distributed between two haplogroups (A and B) were described. Phylogenetic analysis showed that the discovered haplotypes were included in the previously described mountain (Central Caucasian) and lowland (East Caucasian) groups. As can be seen from the median network of haplotypes, the sample from the vicinity of the village of Khasaut is isolated from other mountainous West and Central Caucasian samples and is closest to the East Caucasian lowland samples of the little ground squirrel. Genetic distances obtained by comparing three samples from the Karachay-Cherkess Republic confirm the genetic isolation of the sample from the vicinity of the village of Khasaut. This sample differs from the other two (Uchkulan, Khurzuk) with a distance of 1.54 %. Thus, the results of the conducted study allow us to conclude that in the studied territories (Uchkulan, Khurzuk, Khasaut), the S. pygmaeus species is not homogeneous and is represented by two genetically different groups of the little ground squirrel. Moreover, one of the groups is closer to the East Caucasian (plain), and the other to the Central Caucasian (mountain) samples. The village of Khasaut is located on the left bank of the river, just below the confluence of the Bermamyt River, 6 km from the summit of Bolshoy Bermamyt. According to the data of A.I. Dyatlov et al. (1980), in the vicinity of Mount Bermamyt, mountain little ground squirrels penetrated north of the Rocky Ridge in several places (five settlements), which is not observed in other parts of the range. This is the only place in the gap zone where the rivers do not form barriers between the populations in the mountains and on the plains.

As can be seen from Table 2, of the three studied groups of S. pygmaeus from the Karachay-Cherkess Republic, the Uchkulan and Khurzuk samples are characterized by low values of the haplotype (h = 0.346–0.473) and nucleotide (π = 0.0004–0.0006) diversity indicators. Similar results were obtained earlier for the Central Caucasian samples (Kabardino-Balkarian Republic) of the little ground squirrel, originating from an altitude of 1,200–1,500 m above sea level (Tembotova et al., 2024).

Low genetic diversity in the Elbrus sample was also found by O.A. Ermakov et al. (2023) (haplotype diversity – 0.333 ± 0.215, and nucleotide diversity – 0.03 %). For comparison, we note that for the little ground squirrel groups from the western and eastern lines, the values of the haplotype diversity index varied from 0.859 to 0.964, and the level of nucleotide diversity π varied from 0.17 to 0.76 %, which is almost six or more times higher than in the Elbrus sample (Ermakov et al., 2023). Thus, the results of both the present and previous studies show that most mountain samples of the little ground squirrel in the Western and Central Caucasus (with the exception of the high-mountain Dzhily-Su gorge) are characterized by a low level of genetic diversity (Tembotova et al., 2024). Low values of h and π may be the result of a serious decline in numbers over a long period of time (bottleneck effect) (Kholodova, 2006; Abramson, 2007). It is possible that mountain populations of the little ground squirrel have repeatedly experienced a decline in numbers. Low genetic diversity can lead to a decrease in the adaptive capacity of individuals and populations and increase the risk of their extinction (Gitzendanner, Soltis, 2000; Willi et al., 2006). Relatively high values of the noted indicators were revealed in the sample from the vicinity of the village of Khasaut. Thus, the haplotype diversity was almost two times higher, and the nucleotide diversity was five or more times higher than in the other two samples (Uchkulan, Khurzuk) of the little ground squirrel. The sample from the vicinity of the village of Khasaut is closer in genetic diversity to the previously studied plain samples from the southern edge of the Caspian Lowland of the Eastern Caucasus than to the mountain ones. Such a ratio of genetic diversity indices (high h and π) is characteristic not only of populations that have had a high population size for a long time, but also of those formed as a result of the unification of previously isolated and genetically heterogeneous groups (Rogers, Harpending, 1992).

The significantly negative values of the Tajima’s test observed in almost all of the studied S. pygmaeus samples may indicate a recent population expansion after a decline in numbers (a bottleneck)

The histograms show (Fig. 5) that in the sample from the vicinity of the village of Khasaut, the distribution of nucleotide differences has a multimodal nature, which does not correspond to the expected distribution. Discrepancies between the expected and observed distributions indicate high heterogeneity of the studied sample. In the combined sample (Uchkulan and Khurzuk), the two curves show good agreement and have a unimodal distribution (Fig. 5).

The age of the so-called lowland group (haplogroup A1), which included the mitotypes of the S. pygmaeus from Khasaut, is less than 400 thousand years. The age of clade A2, represented by the ground squirrels of the Eastern Caucasus, is less than 300 thousand years. The group of S. pygmaeus of the Western (Uchkulan, Khurzuk) and Central Caucasus, which form clade B, is phylogenetically younger. Its age is less than 200 thousand years. The calculated age does not contradict the data and opinions of other authors, who believed that the ancestors of modern mountain ground squirrels penetrated into the highlands from lowland areas at different times (Tsvirka, Korablev, 2014). Considering the statements of many researchers (Sviridenko, 1927; Ioff, 1936; Varshavskii, 1963) that in the new history of the range of the little ground squirrel we have to deal not with the primary, but essentially with the repeated dispersal of this rodent, it is likely that the different evolutionary ages of the three identified haplogroups of S. pygmaeus are associated with the multi-stage dispersal of the species across the study area.

The results of molecular dating suggest that the western haplogroup of the little ground squirrel penetrated in a continuous strip into the Central, Eastern Caucasus and the eastern end of the Western Caucasus through the Stavropol Upland and the Caspian Lowland less than 400 thousand years ago

It can also be assumed that as a result of the first stage of settlement, the little ground squirrel became established at the eastern end of the Western Caucasus in the Khasaut area, as well as on the plain and in the foothills of Kabardino-Balkaria, where stable ground squirrel populations existed until 1990 (Tembotov et al., 1969; Tembotova, Kononenko 2017), which have not been registered in the Kabardino-Balkarian Republic since the end of the 20th century. Less than 200 thousand years ago, as a result of settlement, the species rose to the mountains to an altitude of 2,000 m above sea level and higher along the Baksan, Malkinsky and Chereksky gorges. Apparently, it penetrated the border territories of Karachay-Cherkessia along the subalpine belt, as evidenced by the same evolutionary age of animals of the Western (Uchkulan, Khurzuk) and Central Caucasus.

In the Eastern Caucasus, as a result of the first wave of dispersal of the ground squirrel from the Russian Plain, the species was established in the north of the Nogai Steppe (Sukhokumsk) and in the southern outskirts of the Caspian Lowland (Kar-Kar Valley). It is quite clear that the penetration of the little ground squirrel into the southern regions of the Caspian Lowland occurred through the entire Caspian Lowland, based on which it could be assumed that the mitotypes of the species throughout its territory would be of the same evolutionary age. However, given that the Caspian Sea changed its outlines for a very long time in geological time, the lowland was regularly flooded and then freed from water; it is quite understandable that a stable population did not exist there. From the above, it follows that the Caspian Lowland in the areas of Khumtop, Lvov and Zelenomorsk was re-populated by the ground squirrel after 100 thousand years and it is more likely that the settlement came from the Russian Plain.

## Conclusion

The ground squirrel penetrated into the North Caucasus from the western part of a vast range, covering the plain of Eastern Europe, northern Crimea, the Ciscaucasia and the northern parts of Central Asia (Vereshchagin, 1959). The age of the western haplogroup is about 800 thousand years. The spread to the northern parts of Central Asia and the North Caucasus most likely occurred in parallel, since the age of the eastern haplogroup S. pygmaeus 1 and the oldest age of the haplogroups that penetrated into the North Caucasus are close – within 350–400 thousand years for S. pygmaeus 1 and haplogroup A1 (Fig. 2). At the same time, the ground squirrel penetrated into the North Caucasus in a continuous strip to the Central, Eastern Caucasus and the eastern end of the Western Caucasus through the Stavropol Upland and the Caspian Lowland.

As a result of the first wave of settlement, the little ground squirrel became established in the Western Caucasus in the Khasaut area, and in the Central Caucasus, on the plain and in the foothills of Kabardino-Balkaria, where stable populations of ground squirrels existed until 1990 (Tembotov et al., 1969; Tembotova, Kononenko 2017), which have not been recorded in the Kabardino-Balkarian Republic since the end of the 20th century. The absence of a continuous forest belt in the Central Caucasus, in particular in the Kabardino-Balkarian Republic, allowed S. pygmaeus to penetrate into the mountains later, less than 200 thousand years ago, along three gorges: Cherek, Baksan and Malkinsky. It is more likely that the species penetrated into the subalpics of the Western Caucasus (Khurzuk and Uchkulan) from the Central Caucasus

The population in Khasaut is probably a genetic isolate, which is confirmed by genetic distances (within 1.54–1.69 %) (Table 3) with animals from neighboring areas of the Karachay-Cherkess Republic (Uchkulan, Khurzuk) and the Kabardino-Balkarian Republic (Bezengi, Aktoprak, Dzhilysu, Irikchat, Tyrnyauz); however, further research is needed.The first wave of the gopher’s settlement in the Eastern Caucasus (in Dagestan) has survived to this day in the north of the Nogai steppe in the Sukhokumsk region, and in the south of the Caspian lowland in the Kar-Kar1 valley, as evidenced by the evolutionary age of haplogroup A1. The younger age of haplogroup A2 (less than 300 thousand years), also originating from the Eastern Caucasus (Khumtop, Zelenomorsk, Lvovsky 13, Kar-Kar 2), is most likely due to the repeated settlement of the Caspian lowland, regularly flooded by the Caspian waters in historical times. This is also evidenced by the genetic distance (0.76–1.1 %, Table 3) between animals from the Kar-Kar 1 valley and the central regions of the Caspian lowland (Khumtop, Zelenomorsk, Lvovsky 13), which gives reason to believe that the settlement apparently originated from the Russian Plain, and that there is a weak gene flow between these populations, which is probably associated with the low mobility of the species, seasonal movements of its young over short distances (maximum 5 km) (Naumov, 2010), and low numbers.

Regarding the taxonomic status of the Caucasian mountain ground squirrel, we consider it premature to draw any conclusions, since not all territories of the Caucasus were covered by the studies. Nevertheless, the results obtained both in the present study and in the previously conducted one (Tembotova et al., 2024) suggest that the genetic distances (1.33–1.67 %) obtained between the lowland and mountain samples of the little ground squirrel of the North Caucasus correspond only to the level of intraspecific differences, according to the gradation given for the genus Spermophillus by (Baker, Brandley, 2006). Probably, one can agree with the opinion of N.N. Vorontsov and E.A. Lyapunova (1969) that S. musicus is a derivative of S. pygmaeus, made on the basis of karyological studies. M.V. Tsvirka and V.P. Korablev (2014) noted that significant transformations of the karyotype of the mountain ground squirrel occurred after it had already settled in mountainous regions; over time, the characteristics that emerged were fixed, leading to the stable isolation of the Caucasian mountain ground squirrel from the lowland populations of the little one. The genetic differentiation and structuring that we observed in the S. pygmaeus species in the conditions of the North Caucasus are probably also due to the geographical isolation of the lowland and mountain populations, which caused the emergence of local adaptations to habitat conditions as a result of a reduction in numbers and fragmentation of the range, which is still observed today.

## Conflict of interest

The authors declare no conflict of interest.
